# The Laws of Health

**Published:** 1890-01

**Authors:** 


### THE LAWS OF HEALTH.

In the course of the yearly oration in memory of Harvey, the discoverer of the circulation of the blood, at the Royal College of Physicians in England, Dr. James E. Pollock made the interesting statement, based on statistics, that an average of two years had been added to the life of each male born in England and Wales, and nearly 3½ years to the lifetime of every female born, by the reduction of the death rate, and that this was largely due to increased medical knowledge and the greater recognition of the natural laws of health and disease. He further stated that 70 per cent. of the years thus added to the lives of males during the 1876–80 were lived at the usual ages between 20 and 60, whilst of the remainder 22 per cent. were lived under 20 years, and 8 per cent above 60 years.

